# The relationship between psychosocial working conditions and sickness absence days among employees reporting symptoms of common mental disorders in Germany

**DOI:** 10.1007/s00420-026-02205-7

**Published:** 2026-03-11

**Authors:** Meike Heming, Florian Angerer, Christoph Kröger, Gianni Lidolt, Nicole R. Hander, Eva Rothermund, Harald Gündel, Nadine Mulfinger, Ute Schröder, Uta Wegewitz, Regina Herold, Peter Angerer

**Affiliations:** 1 Institute of Occupational, Social, and Environmental Medicine, Centre for Health and Society, Medical Faculty and University Hospital Düsseldorf, Heinrich-Heine-University Düsseldorf, Moorenstraße 5, 40225 Düsseldorf, Germany; 2 Institute of Psychology, University of Hildesheim, Universitätsplatz 1, 31141 Hildesheim, Germany; 3 Department of Psychosomatic Medicine and Psychotherapy, Ulm University Medical Center, Albert-Einstein-Allee 23, 89071 Ulm, Baden-Württemberg Deutschland; 4 Department of Psychiatry II, Ulm University & BKH Günzburg, Günzburg, Germany; 5 Division 3 Work and Health, Federal Institute for Occupational Safety and Health, Nöldnerstraße 40-42, 10317 Berlin, Germany; 6 Department of Psychosomatic Medicine and Psychotherapy, University Hospital of Erlangen, Friedrich-Alexander University Erlangen-Nürnberg, Erlangen, Germany

**Keywords:** Working conditions, Mental health, Sick leave, Occupational stress, Workplace, Mental disorders

## Abstract

**Purpose:**

Psychosocial working conditions influence the risk for sickness absence days. These relationships can differ for employees with common mental disorders (CMDs). This study therefore investigates relationships between psychosocial working conditions and sickness absence days in employees reporting CMD symptoms.

**Methods:**

Data of participants of a randomized controlled trial are investigated (*n* = 529). The Copenhagen Psychosocial Questionnaire assessed 12 scales of working conditions at baseline. The outcomes number of sickness absence days were assessed nine and 15 months later, as well as during the follow-up period. Negative binomial regression analyses were adjusted for several factors and were conducted for imputed data and supplemented by complete case analyses.

**Results:**

Influence at work was associated with lower risks of sickness absence days nine (RR= -0.009, CI= − 0.018; − 0.001) and 15 months later (RR= − 0.008, CI= − 0.015; 0.000). Dissolution (i.e., reporting to work outside of work hours or being available for colleagues during free time) was associated with lower risks of sickness absence days during the follow-up period. Complete case analyses showed positive associations between social support and sickness absence days nine (RR = 0.01, CI = 0.0; 0.02; *n* = 377) and 15 months later (RR = 0.01, CI = 0.0; 0.03; *n* = 320).

**Conclusions:**

Among employees reporting symptoms of CMDs, influence at work and dissolution predicted sickness absence days for different time periods. However, many psychosocial working conditions showed no such relevance, indicating that other factors may be more relevant in regard of sickness absence days among this particular group of employees.

**Trial registration:**

The randomized controlled trial was registered at the German Clinical Trial Register on 01.03.2021 (DRKS00023049; https://drks.de/search/en/trial/DRKS00023049).

**Supplementary Information:**

The online version contains supplementary material available at 10.1007/s00420-026-02205-7.

## Introduction

At some point in their lives, almost 30% of individuals worldwide experience a common mental disorder (CMD, i.e., depression, anxiety- or stress-related disorder) (Steel et al. 2014). One in five adults experiences a CMD within the past 12 months (Steel et al. 2014). CMDs further belong to the most common and cost-intensive diseases in Germany (Storm et al. 2023). The presence of CMD can lead to impairments that result in sickness absence days and thus represent an important cause for sickness absence days (Montano 2020). CMDs also lead to longer durations of sickness absence days and increase the risk for disability pension in Germany (Amiri and Behnezhad 2019; Badura et al. 2023; Storm et al. 2023). For example, 16.7% of all sickness absence days in one year are due to CMDs (Klemm et al. 2024). The duration of sick leave per case of CMD is substantially higher than for other diseases and accounts for 38 days on average (Klemm et al. 2024). This indicates the need for research that investigates the factors that can enable or hinder employees suffering from CMDs to stay at work. As determinants for sickness absence are complex and can be multidimensional (Montano 2020), it is important to consider the workplace and the possible impact of psychosocial working conditions (Niedhammer et al. 2017). Psychosocial working conditions describe a combination of psychological and social factors connected to the work environment. Today, there are numerous stress-theoretical models available which assess different psychosocial working conditions and explain their associations towards stress and disease in working populations. One of the most prominent models is the job-demand-control-support model (Karasek 1979). It assumes that when demands at work are high (such as high workload or time pressure), control and support from colleagues or superiors is low (such as having no say in decisions regarding works tasks or receiving help from colleagues), work stress and ultimately diseases can arise (Karasek 1979). Associations between psychosocial working conditions and sickness absence have been extensively investigated to date and have shown either increased or reduced risks of sickness absence (Bertrais et al. 2023; Holmgren et al. 2009; Niedhammer et al. 1998; Slany et al. 2014; Taibi et al. 2021). For example, high demands or low work-life balance were found to be risk factors for the occurrence of sickness absence among workers of 31 European countries (Niedhammer et al. 2013). Reporting high levels of decision authority/job control predicted lower absence rates among Belgian and Danish workers (Nielsen et al. 2004). A meta-analysis also showed that the combination of job demands and control (i.e., job strain) was associated with subsequent sick leave (Amiri and Behnezhad 2020). Using the Copenhagen Psychosocial Questionnaire (COPSOQ), which covers a wide range of different psychosocial working conditions, it was shown that high quantitative demands, low sense of community, low social support or low quality of leadership, for example, are associated with more than seven days per year of sickness absence among European workers (Slany et al. 2014).

However, less research on these associations is available among the group of individuals who experience CMDs (but are still actively working). Therefore, mechanisms that may explain the associations between working conditions and sickness absence days among employees with CMD are rare. Next to the job-demand-control-support model and its sound evidence base, there may be many other factors beyond working conditions, e.g. factors on the level of the organization and non-work related factors on the level of family, community or national laws. They all are relevant for sickness absence among individuals suffering from CMD symptoms. While the above mentioned job-demand-control-support model postulates that the experience of intense job strain may result in disorders over long-term, a different picture can emerge for individuals, already experiencing symptoms: These individuals may have, according to the stress-vulnerability model, a higher vulnerability due to their symptoms or other predispositions, reaching more easily their own stress-threshold or changing the psychological reaction to stress (Zubin and Spring 1977). Thus, their perception of working conditions, can potentially show different associations with sickness absence days, than for healthy employees. In addition, it may be possible that other factors, outside of the working environment, also have an effect on sickness absence. The psychosocial theory of sick leave suggests that the subjective perception of the health status or self-efficacy is relevant for the likelihood of being on sick leave (Montano 2020). Experiencing stressors outside of the work environment, such as family conflicts or other personal reasons can also affect sickness absence (Montano 2020).

Among Swedish employees with CMDs, it was recently shown that low control, job strain, no flexible work hours or possibilities to work from home were associated with subsequent higher risks of sickness absence days (Helgesson et al. 2023). A scoping review focusing on employees with CMDs indicated that working conditions such as high job demands, low job control or high job strain were predictors of sickness absence (de Vries et al. 2018). Poor support from a superior was a determinant for sickness absence in workers with CMD in Norway (Foss et al. 2010). A systematic review covered 13 prospective studies and evaluated relationships between psychosocial stressors and risk of sickness absence due to a diagnosed mental disorder (Duchaine et al. 2020). Low reward, effort-reward imbalance and job strain showed the highest risk for sickness absence, while high demands and low control were also separately associated with higher risks of sickness absence (Duchaine et al. 2020). Of the studies evaluated within the two reviews, none were conducted among workers in Germany (de Vries et al. 2018; Duchaine et al. 2020). As countries have different regulations regarding sick leave at work, social insurance systems, labor law and also have different health systems with other treatment options for CMD, it is important to also generate evidence for employees in Germany. For example, treatment can be performed by in- or outpatient care, can be provided by psychiatrists or psychological psychotherapists and funding modalities can differ (i.e., mandatory health insurance or pension insurance) (Salize et al. 2007; Sikora et al. 2019). In Germany, employees get salary for the first six weeks of sickness absence and labor law states that physician certified sickness absence needs to be provided after the third calendar day of sickness absence. After six weeks, the statuary health insurance provides money which is somewhat less than the salary. After six weeks of sickness absence per year a company must offer support for the return to work process. Although it is also mandatory for companies in Germany to conduct psychosocial risk assessments, these are only rarely implemented yet (Beck and Lenhardt 2019). Therefore, knowledge on the determinants is important to find starting points for prevention approaches aimed at reducing the number of sick days and helping employees with CMDs to stay at work. Therefore, this study can be differentiated from previous prospective studies which investigated, for example, sickness absence due to CMD (Duchaine et al. 2020).

In addition to psychosocial factors of the working environment, there are also other (personal) factors, such as sex, age, occupational level or the past history of absenteeism that can have an impact on sickness absence days among employees with CMDs (de Vries et al. 2018). In general, women tend to have higher numbers of sickness absence days due to CMDs than men (Storm et al. 2023). However, some studies suggest that these differences may be explained by the profession itself (as women are predominantly employed in healthcare, educational or social sectors, where emotional demands and direct contact to patients/clients are high) (Laaksonen et al. 2010; Lidwall 2021; Timp et al. 2024). The proportion of sickness absence days due to CMDs also increases with age and remains relatively stable from the age of 40, which underlines the importance of CMDs for middle-aged and older employees (Storm et al. 2023). The occupational level (blue vs. white collar) was associated with long-term sickness absence in individuals with adjustment disorder (Catalina-Romero et al. 2012). Thus, these factors should be acknowledged when investigating psychosocial working conditions and sickness absence.

To sum up, there is a research gap regarding psychosocial working conditions and their relationship with sickness absence days among employees with CMDs, especially in Germany. Therefore, this study aims to investigate several psychosocial working conditions in a group of employees reporting symptoms of CMDs and participating in a randomized controlled trial investigating effectiveness of psychotherapeutic consultation at work (PT-A), taking into account personal characteristics. The aim is to investigate which psychosocial working conditions can predict sickness absence days in this specific study group over different time periods.

## Methods

### Study design and study sample

Data of study participants (n = 549) in a randomized controlled trial called *friaa* (in German: „Frühe Intervention am Arbeitsplatz”) were investigated in this study (Weber et al. 2021). *Friaa* investigates the effectiveness of psychotherapeutic consultation at work by providing a modular-based intervention to employees who suffer from CMDs or symptoms of CMDs. Inclusion criteria were an age of at least 18 years, being employed for at least 15 h per week and reporting symptoms or a diagnosis of CMDs at study begin. If no subclinical symptoms were reported, the psychotherapists used the Global Assessment of Functioning Scale (GAF) within the first diagnostic session and included participants with a score lower than 81 (Hall 1995). Exclusion criteria were diagnoses of psychosis, substance abuse, schizophrenia, severe somatic health problems, current psychotherapeutic treatment or being an applicant for retirement pension (Weber et al. 2021). Participants were mainly recruited at five study centers in Germany, and three study centers additionally recruited via social media. All participants received a diagnostic session with a psychotherapist before they were randomly allocated to intervention or control group. Participants in the intervention group could receive up to 16 sessions in total within a period of nine months. Participants in the control group received recommendations for care as usual and a subsequent supportive phone call. A detailed description of the study design can be found elsewhere (Weber et al. 2021). Ethical approval was obtained from the ethic committees of the study centers: Ulm University (November 2020, 339/20), Friedrich-Alexander University Erlangen-Nürnberg (December 2020, 525_20 Bc), University of Hildesheim (January 2021, 165) and Heinrich-Heine-University Düsseldorf (January 2021, 2021–1279) (Weber et al. 2021). Participants gave written informed consent.

Data collection at different time points for the relevant study variables took place from September 2021 to May 2024. Participants completed online questionnaires at three time points: at baseline before receiving the intervention (T0), nine months later after the intervention (T1), and further six months later (T2). At T0, 549 participants completed the questionnaires. At T1, 420 participants completed the questionnaires and at T2, 372 participants completed the questionnaires. There were some mandatory questions within the online entry mask that had to be answered in order to continue and complete the questionnaires.

### Measures

**Fig. 1:**
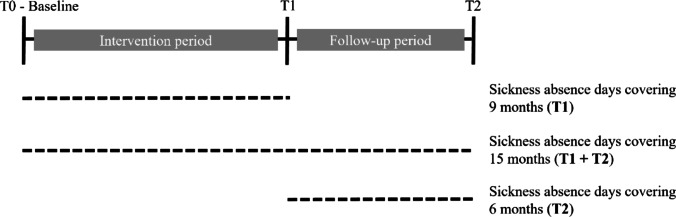
Description of periods covering sickness absence days as the three study outcomes

Sickness absence days were assessed at T0, T1 and T2 using two items. The first item asked whether participants have been absent from work due to health reasons (yes/no). For T0 and T2 this was asked for the past six months, and for T1 this was asked for the past nine months (i.e., intervention period). If participants answered this first item with a yes, they were asked to report the number of sickness absence days for the respective time period. Answers of participants which indicated to not have sickness absence days in the first item at the respective time point, were recoded as a zero for the second item to be able to also show zero sickness absence days. Thus, higher values indicate higher sickness absence days. In analyses, this recoded count variable for sickness absence days was used. Further, sickness days of T1 and T2 have been added up to reflect sickness absence days for the overall study period covering 15 months. Assessed were three different outcomes for sickness absence days: at first, nine months after baseline (i.e., period in which the intervention took place; T1), at second, 15 months after baseline (i.e., period of intervention plus follow-up period; T1 + T2), and at third, six months after intervention (i.e., only follow-up period, T2). Please see Fig. 1 for a visualization of periods covering sickness absence days. Due to the early stage of disease within the study population at hand, all sickness absence days were investigated. It may be expected that the more severe the CMD symptoms becomes, the more sickness absence days are reported. Especially in the beginning of developing symptoms it may be that employees use few sickness absence to recover from or cope with their symptoms (Lexis et al. 2009). CMDs may also lead to physical symptoms and can be accompanied by physical diseases indicating the relevance of including all sickness absence days.

Psychosocial working conditions were assessed at T0 with 12 scales of the third German version of the validated Copenhagen Psychosocial Questionnaire (COPSOQ) (Lincke et al. 2021). The scales were chosen as relevant items addressing psychosocial conditions at work as they can reflect components of the above-mentioned stress-theoretical constructs. Three scales were used to measure demands indicating good and questionable reliability (for Dissolution): Quantitative demands (five items, Cronbach’s alpha = 0.80), emotional demands (two items, Cronbach’s alpha = 0.84) and dissolution (two items, Cronbach’s alpha = 0.63). An example item for quantitative demands is “Do you get behind with your work?”, for emotional demands “Is your work emotionally demanding?” and for dissolution “I take care of work-related tasks outside of my working time as well.” (Lincke et al. 2021; Llorens et al. 2019). To assess influence and possibilities for development three scales were used indicating questionable and acceptable reliability: Influence at work (two items, Cronbach’s alpha = 0.62), degrees of freedom (Breaks at work; one item) and possibilities for development (three items, Cronbach’s alpha = 0.74). Example items are: “Can you influence the amount of work assigned to you?” (Influence at work), “Can you decide when to take a break?” (Degrees of freedom) and “Do you have the possibility of learning new things through your work?” (Possibilities for development) (Lincke et al. 2021). To assess social relations and leadership six scales were used indicating acceptable and good reliability: Quality of leadership (four items, Cronbach’s alpha = 0.87), support at work (four items, Cronbach’s alpha = 0.79), sense of community (two items, Cronbach’s alpha = 0.86), unfair treatment (one item), trust and justice (four items, Cronbach’s alpha = 0.73), and recognition (one item). Example items for the six scales are the following: “To what extent would you say that your immediate superior gives high priority to job satisfaction?” (Quality of leadership), “How often do you get help and support from your colleagues, if needed?” (Support at work), “Is there a good atmosphere between you and your colleagues?” (Sense of community), “How often do you feel unjustly criticised, bullied or shown up in front of others by your colleagues or your superior?” (Unfair treatment), “Are conflicts resolved in a fair way?” (Trust and justice) and “Is your work recognized and appreciated by the management?” (Recognition) (Lincke et al. 2021). Answers were given on a five-point Likert scale going from one always to five = never or hardly never or from one = to a very large extent to five = to a very small extent. Answers have been recoded to a five point scale from zero to 100 and mean scales were computed if at least half of the items of each scale were answered by the participants (Lincke et al. 2021). All items were recoded in a way that higher values indicate higher levels of the measured criterion (i.e., higher demands, higher influence at work).

Some covariables were assessed and used for adjustment within analyses: sex (female/male), age (continuously measured), occupational position was dichotomized to indicate blue or white-collar workers (Weber et al. 2024) and treatment group (control group/intervention group). However, results of the friaa RCT could not show significant intervention effects for the primary outcome being sickness absence days at 15 months (Rothermund-Nassir 2025).

Furthermore, we have adjusted for symptoms of CMDs with calculated sum scores of complete cases of depression severity (Patient-Health-Questionnaire; PHQ-9; (Löwe et al. 2002), anxiety symptoms (Generalized Anxiety Disorder Scale-2; GAD-2; (Kroenke et al. 2007) and somatic symptoms (Somatic Symptom Scale – 8; SSS-8; (Gierk et al. 2014). Cronbach’s alpha for depression severity was 0.77 at T0 and 0.87 at T1, which indicates acceptable and good internal reliability. The PHQ-9 assesses the frequency of criteria for depression of the past two weeks with nine items ranging from zero to 27. Scores between five and 10 indicate mild symptoms, while scores below five usually indicate the absence of a depressive disorder (Löwe et al. 2002). Cronbach’s alpha for anxiety symptoms was 0.75 at T1 and 0.8 at T2, which indicates acceptable and good internal reliability. The GAD-2 assesses anxiety symptoms of the past two weeks with two items ranging from zero to six and indicates clinically relevant anxiety symptoms with scores greater than or equal to three (Kroenke et al. 2007). Cronbach’s alpha for somatic symptoms was 0.72 at T1 and 0.83 at T2, which indicates acceptable and good internal reliability. The SSS-8 assesses somatic symptom burden of the past seven days with eight items rangingfrom zero to 32. Scores between four and seven indicate low and scores between eight and 11 indicate medium somatic symptom burden, while a high somatic burden usually is identified with a cut-off value equal or greater than 12 (Gierk et al. 2014).

As this study aims to present findings for individuals with symptoms of CMDs, participants who did not report relevant symptoms of the respective scales were excluded from the analyses (*n* = 18): We chose a cut-off of five for depression severity, a cut-off of three for anxiety symptoms and a cut-off of eight for somatic symptoms. Thus, the results of the imputed data are shown for *n* = 529.

### Statistical analyses

Descriptive statistics are shown for the study variables and characteristics of the study sample with mean, standard deviation (*SD*) or minimum and maximum (min and max). In negative binomial regression analyses, it was investigated which psychosocial working conditions predict sickness absence days at T1, T1 plus T2, and T2 (Fig. 1). In a first step, one of the periods for sickness absence days is chosen as the outcome variable, and sex, age, treatment group, occupational position, sickness absence days and symptoms of CMDs are included for adjustment. Each measured scale is separately included to investigate the impact of each single psychosocial working condition (this step is only reported in the supplementary information, ESM_1, ESM_2). In a next step, all scales are included into one model to investigate which of the scales has the strongest association with sickness absence days. Analyses are adjusted for baseline values. In case of T2 analyses, sickness absence days and symptoms of CMDs are adjusted for values at T1.

To address missing data, we employed multiple imputations (MI), which is a state-of-the-art procedure to handle incomplete data commonly appearing in RCTs (Enders 2022). The imputation procedure was implemented according to recommendations of Enders (2022). We generated 15 imputed datasets, each created using 50 iterations to ensure convergence of the imputation algorithm. Missing data were imputed through predictive mean matching, which is appropriate for continuous variables and is relatively robust against departures from normality in the distribution of the data. The imputation model included all variables used in the subsequent analyses, as well as auxiliary variables that might predict missingness, to improve the plausibility of the MAR assumption. To match the analysis strategy, data were imputed in wide format as single level MI, which is adequate for studies with a balanced longitudinal design (Grund et al. 2019). Following imputation, we fitted regression models to each of the 15 imputed datasets separately. Results from these analyses were then pooled using Rubin’s rules (Rubin 1987) to account for both within- and between-imputation variability. To assess the sensitivity of our results to the missing data handling approach, we also conducted complete case analyses, which are reported in the supplementary information (ESM_3) to provide transparency and allow for comparison. As some questionnaires were mandatory to fill in for the participants (such as depressive symptoms or sickness absence days), our missing data mainly indicate non-participation in the follow-up questionnaires. At T1, we observe that about 72% of participants have complete cases for our study variables. For example, at baseline this is the case for about 93% of participants.

Analyses were conducted with R Version 4.4.1. Negative binomial regression analyses were calculated using the MASS package (Venables and Ripley 2002) with the glm.nb function. Multiple imputations and pooling of the regression models were implemented using the mice package (van Buuren and Groothuis-Oudshoorn 2011). 95% confidence intervals (CI) and rate ratios (RR) are shown. RR are obtained by exponentiating the regression coefficients and represent the factor by which the number of predicted sickness absence days increases or decreases for a one-unit change in the predictor, respectively. Statistical significance was assumed at a level of *p* < 0.05. For model fit statistics, we report Akaike Information Criterion (AIC), Bayesian Information Criterion (BIC) and MC Fadden’s *R*^2^ (McFadden 1972).

## Results

### Study sample

One person reported a diverse gender and had to be excluded from analyses, and one person had missing data for all variables (*n* = 547). Further 18 participants were excluded due to insufficient symptom reporting (see measures section), resulting in a study sample of *n* = 529 for imputed data.

### Study characteristics

Table 1 shows descriptive statistics for the study variables (*n* = 529). Approximately half of the participants were female (55%) and the mean age was 46 years. A relatively small number of participants were blue-collar workers (23%). Participants reported an average of 23 days of sickness absence at baseline (T0), 58 days over the entire study period (T1 + T2) and 37 days during the intervention period (T1). In terms of symptom severity, participants reported on average clinically relevant anxiety symptoms, as well as a moderate depression and a high somatic symptom burden. Of the sample, almost 87% had an ICD-10 diagnosis, assessed by the study psychotherapist in the first session. In addition, participants reported, for example, a mean value of 68.9 (*SD* = 30.49) for degrees of freedom regarding breaks at work and a mean value of 39.62 (*SD* = 25.23) for quality of leadership.

**Table 1:** Descriptive statistics of the study sample at baseline (*n* = 529)

	*n* (%) or min-max	Mean	SD^a^
**Sex**			
Female	293 (55)		
Male	236 (45)		
**Age** (years)	21–64	46.01	11.04
**Treatment group**			
Intervention group	270 (51)		
Control group	259 (49)		
**Occupational position**			
Blue-collar workers	120 (23)		
White-collar workers	409 (77)		
**Sickness absence days**	0–187	22.61	33.46
**Sickness absence days T1 + T2** (*n* = 338)	0–450	58.07	90.69
**Sickness absence days T1** (*n* = 403)	0–270	36.57	61.41
**Sickness absence days T2** (*n* = 352)	0–200	22.32	41.08
**Common mental disorders** ^**b**^			
Depression severity symptoms	2–27	13.24	4.87
Anxiety symptoms	0–6	3.53	1.69
Somatic symptoms (*n* = 513)	1–30	13.43	5.49
**Psychosocial working conditions** ^**c**^ (5-point scale from 0 to 100; *n* = 513–529)			
**Demands**			
Quantitative demands	0–100	55.51	21.51
Emotional demands	0–100	48.93	32.54
Dissolution	0–100	34.14	27.01
**Influence and possibilities for development**			
Influence at work	0–100	40.05	23.21
Degrees of freedom	0–100	68.9	30.49
Possibilities for development	0–100	62.95	21.38
**Social relations and leadership**			
Quality of leadership	0–100	39.62	25.23
Support at work	0–100	58.83	22.61
Sense of community	0–100	70.71	21.33
Unfair treatment	0–100	26.85	28.06
Trust and justice	0–100	56.54	17.25
Recognition	0–100	42.85	27.96

^a^ Standard deviation

^b^ Participants were included when above the chosen cut-off values for reported symptoms: cut-off of five for depressive symptoms, cut-off of three for anxiety symptoms and cut-off of eight for somatic symptoms

^c^ Higher values indicate higher levels of the measured criterion

### Results from regression analyses

Table 2 shows results of regression analyses estimating sickness absence days at T1 for imputed data (*n* = 529). Of the 12 included COPSOQ scales, only one shows a significant negative association with sickness absence days for the intervention period, when controlled for several potential confounders. Participants who report to have higher influence at work, report less sickness absence days at T1. For every one-unit increase on the scale (ranging from one to 100) for influence at work, there is a 0.09% decrease in sickness absence days at T1 (RR = 0.991, CI = 0.982; 0.999, Table 2). Analyses considering each psychosocial working condition scale separately show similar results, with only one significant negative association between influence at work and sickness absence days at T1 (ESM_1, ESM_2).

**Table 2:** Results for negative binomial regression analyses estimating the association between psychosocial working conditions and sickness absence days at T1 (imputed data, *n* = 529)

	Sickness absence days T1			
		95% CI^a^		
	RR^b^	lower	upper	*p*-value
Sickness absence days T0	**1.014**	**1.009**	**1.018**	**0.000**
Sex	0.855	0.633	1.156	0.307
Age	**1.024**	**1.006**	**1.043**	**0.009**
Treatment group	1.099	0.788	1.532	0.575
Occupational position	0.829	0.537	1.278	0.392
Depression severity	**1.061**	**1.007**	**1.118**	**0.026**
Anxiety symptoms	1.020	0.915	1.138	0.720
Somatic symptoms	0.997	0.959	1.035	0.861
**Demands**				
Quantitative demands	1.002	0.992	1.011	0.726
Emotional demands	0.998	0.992	1.003	0.461
Dissolution	1.002	0.995	1.008	0.650
**Influence and** **possibilities for development**				
Influence at work	**0.991**	**0.982**	**0.999**	**0.032**
Degrees of freedom	1.001	0.995	1.008	0.644
Possibilities for development	1.003	0.993	1.014	0.505
**Social relations and leadership**				
Quality of leadership	0.996	0.986	1.006	0.410
Support at work	1.009	0.996	1.021	0.175
Sense of community	0.997	0.989	1.006	0.532
Unfair treatment	1.003	0.996	1.010	0.337
Trust and justice	1.001	0.989	1.013	0.877
Recognition	0.999	0.991	1.006	0.714
**Model Fit**	AIC^c^	BIC^d^	MC Fadden’s *R*^2^	
	4473.310	4567.272	0.024	

^a^ 95% confidence interval

^b^ Rate ratio

^c^ Akaike information criterion

^d^ Bayesian information criterion

Bold values indicate a p-level below 0.05

Table 3 shows results of regression analyses estimating sickness absence days for the whole study period (i.e., T1 + T2) for imputed data (*n* = 529). Similar to the results observed in the T1 analyses, influence at work is negatively associated with sickness absence days. Participants who report higher influence at work, show less sickness absence days over the entire study period. Each one-unit increase on the influence at work scale is associated with a 0.08% decrease in days of sickness absence (RR = 0.992, CI = 0.985; 1.000, Table 3). Analyses considering each psychosocial working condition scale separately, show similar results and only one significant negative association between influence at work and sickness absence days for the whole study period (ESM_1, ESM_2).

**Table 3:** Results for negative binomial regression analyses estimating the association between psychosocial working conditions and sickness absence days for the whole study period (imputed data, *n* = 529)

	Sickness absence days T1 + T2			
		95% CI^a^		
	RR^b^	Lower	Upper	*p*-value
Sickness absence days T0	**1.011**	**1.007**	**1.015**	**0.000**
Sex	0.913	0.692	1.205	0.519
Age	**1.025**	**1.009**	**1.042**	**0.002**
Treatment group	0.996	0.755	1.314	0.979
Occupational position	0.815	0.544	1.221	0.317
Depression severity	1.037	0.993	1.082	0.098
Anxiety symptoms	1.029	0.933	1.136	0.563
Somatic symptoms	1.013	0.979	1.048	0.451
**Demands**				
Quantitative demands	0.999	0.991	1.006	0.702
Emotional demands	0.998	0.992	1.003	0.369
Dissolution	0.999	0.994	1.005	0.759
**Influence and** **possibilities for development**				
Influence at work	**0.992**	**0.985**	**1.000**	**0.037**
Degrees of freedom	1.001	0.995	1.006	0.843
Possibilities for development	1.006	0.997	1.015	0.208
**Social relations and leadership**				
Quality of leadership	0.995	0.986	1.004	0.288
Support at work	1.007	0.997	1.017	0.171
Sense of community	0.994	0.987	1.002	0.158
Unfair treatment	1.003	0.997	1.009	0.300
Trust and justice	1.003	0.992	1.014	0.572
Recognition	0.999	0.992	1.006	0.852
**Model Fit**	AIC^c^	BIC^d^	MC Fadden’s *R*^2^	
	5130.099	5224.061	0.023	

^a^ 95% confidence interval

^b^ Rate ratio

^c^ Akaike information criterion

^d^ Bayesian information criterion

Bold values indicate a p-level below 0.05

Table 4 shows results of regression analyses estimating sickness absence at T2 for imputed data (*n* = 529). Results show that different psychosocial working conditions are associated with sickness absence days during the follow-up period. Participants who report higher dissolution (i.e., reporting to also work outside of work hours or being available for colleagues in their free time), report less sickness absence days at T2. For each one-unit increase on the dissolution scale, sickness absence days at T2 decreased by 0.07% (RR = 0.993, CI = 0.986; 1.000, Table 4). Analyses considering each psychosocial working condition scale separately show similar results, with only one significant negative association between dissolution and sickness absence days at T2 (ESM_1, ESM_2). Due to convergence issues, support at work, unfair treatment and trust and justice had to be excluded as predictors in these analyses to ensure model stability.

**Table 4:** Results for negative binomial regression analyses estimating the association between psychosocial working conditions and sickness absence days at T2 (imputed data, *n* = 529)

	Sickness absence days T2			
		95% CI^a^		
	RR^b^	Lower	Upper	*p*-value
Sickness absence days T1	**1.011**	**1.008**	**1.014**	**0.000**
Sex	1.102	0.795	1.529	0.559
Age	1.014	0.998	1.031	0.093
Treatment group	0.893	0.621	1.285	0.539
Occupational position	0.835	0.532	1.308	0.427
Depression severity T1	1.033	0.975	1.094	0.274
Anxiety symptoms T1	0.953	0.801	1.134	0.584
Somatic symptoms T1	1.042	0.998	1.089	0.060
**Demands**				
Quantitative demands	0.995	0.986	1.005	0.349
Emotional demands	1.000	0.994	1.006	0.984
Dissolution	**0.993**	**0.986**	**1.000**	**0.042**
**Influence and** **possibilities for development**				
Influence at work	0.997	0.988	1.006	0.528
Degrees of freedom	0.998	0.992	1.003	0.405
Possibilities for development	1.011	0.999	1.022	0.065
**Social relations and leadership**				
Quality of leadership	1.000	0.991	1.008	0.923
Support at work				
Sense of community	0.997	0.989	1.005	0.474
Unfair treatment				
Trust and justice				
Recognition	1.000	0.993	1.008	0.915
**Model Fit**	AIC^c^	BIC^d^	MC Fadden’s *R*^2^	
	3742.765	3823.914	0.040	

^a^ 95% confidence interval

^b^ Rate ratio

^c^ Akaike information criterion

^d^ Bayesian information criterion

Bold values indicate a p-level below 0.05

### Results from regression analyses for complete cases

In the supplementary information (ESM_3) results are presented for complete case analyses. Within these analyses, only participants with no missing values for the respective measures were considered resulting in samples of *n* = 377 for T1, *n* = 320 for T1 plus T2, and *n* = 313 for T2 analyses. Obtained results are similar and supportive of the imputed data analyses, but show additional potential relevant psychosocial working conditions for sickness absence days: Participants reporting higher social support from superiors or colleagues showed higher sickness absence days at T1 and at the whole study period (T1 + T2, ESM_3). Further, complete case analyses showed a positive association between possibilities for development and sickness absence days at T2 as well as a negative association between sense of community and sickness absence days at T2 (ESM_3). The association between influence at work and sickness absence days for the whole study period for imputed data is not observed in complete case analyses (ESM_3).

## Discussion

This study aimed to investigate whether psychosocial working conditions are predictors of sickness absence days in a sample of employees reporting symptoms of CMDs and participating in a randomized controlled trial investigating effectiveness of psychotherapeutic consultation at work (PT-A). Results showed that higher influence at work at baseline predicted a lower risk of sickness absence days at nine (i.e., intervention period) and 15 months later (i.e., intervention and follow-up period). Dissolution was negatively associated with sickness absence days during the follow-up period (six months after intervention). In sensitivity analyses, complete case analyses showed positive associations between social support and sickness absence days nine and 15 months later (ESM_3).

We have shown that employees who report higher influence at work, report less sickness absence days nine months later (intervention period) and 15 months later (intervention period plus follow-up period). Thus, we can suggest that out of several investigated psychosocial working conditions, for employees with symptoms of CMDs, influence at work may be relevant for reduction of sickness absence days. This supports findings in the literature, stating that high job control predicted lower risk of long-term sickness absence due to depressive disorders among male Japanese workers (Inoue et al. 2010). However, these workers had no history of previous mental disorders at baseline (Inoue et al. 2010). Other research showed that low job control predicted long-term sickness absence (more than 28 days) due to depression in both men and women, also after adjustment for baseline depressive symptoms (Clumeck et al. 2009). In Swedish employees seeking primary health care, it was also shown that low influence at work was associated with subsequent register-based sickness absence days of more than 14 days (Hultén et al. 2022). Investigation of employees with diagnosed CMDs showed as well that low control at baseline was predictive of increased risks of sick leave (more than 14 days) three years later (Helgesson et al. 2023). Thus, our findings are in line with the suggestion of the job-demand-control-support model, that high control at work can reduce or even buffer negative consequences (Karasek 1979).

In analyses covering the follow-up period (i.e., six months after intervention), dissolution was predictive of lower sickness absence days. This rather counterintuitive finding may be explained. The scale dissolution of work and private life is a newer edition to the third version of the COPSOQ, which showed questionable Cronbach’s alpha values (Lincke et al. 2021). Dissolution was associated with the inability to relax and with presenteeism (Lincke et al. 2021). There is the possibility that the scale measures slightly different aspects of dissolution. The items are worded in a way that they ask about working outside of normal working hours and being available for colleagues during free time, but it may also be possible that individuals understand these items in a way that it would reflect flexible work arrangements and thus the possibility to work at their own pace or time. If this would be true, employees reporting symptoms of CMDs can potentially manage their work better, if they are given more flexibility by their employer. This was for example shown by Helgesson et al. (2023) who found that no flexibility regarding work hours or no possibility to work from home were predictive of increased sickness absence days. In contrast to this explanation, it could also show presenteeism, indicating that the employees are still working while being ill (Bernstrøm and Houkes 2018). However, as we could not find similar results for this association in the two other study periods, the finding at hand could also have occurred by chance and should be considered with caution. In addition to this, it may be worth mentioning, that all participants reported on average less symptoms of CMDs after the intervention period compared to baseline (data not shown). This may indicate, that when symptoms are less severe, employees may be more likely to avoid taking sick leave, when they have more flexible work arrangements (e.g., working at own time or pace). Further investigations are warranted to support this suggestion. In addition, the analyses showed that the assessed symptom severity was not predictive of sickness absence days at any time point (only exception was depression severity at nine months). Other research findings showed more consistently that a high CMD symptom severity is a predictor of sickness absence days (de Vries et al. 2018). As the present study population had, for example, on average a high somatic burden or moderate depression severity, this may suggest that some of the psychosocial working conditions, such as influence at work, are relevant predictors for sickness absence days in this specific study sample.

Similar to Helgesson et al. (2023), we have not found associations between support at work and sickness absence days. However, we did observe positive associations between support at work and sickness absence days after nine and 15 months in sensitivity analyses when considering complete case analyses, showing that social support was associated with higher risks of subsequent sickness absence days (ESM_3). This may indicate that only among the full observations, the perception of having supportive colleagues or superiors may encourage them to take sick leave or make them feel less pressured to attend work when being sick, resulting in more sickness absence. Thus, in this case this may be a proxy for a supportive work environment. This line of thought is also supported by a study finding that high emotional support was associated with more sickness absence spells and days in a general middle aged working population in Sweden suggesting that this reflects an illness behavior (Karlsson et al. 2010). As that study did not focus on CMDs in particular, this behavior seems to rather fit to general sickness absence, caused by different diseases. However, as findings for imputed and complete data were different in this case and as single analyses for psychosocial working conditions could not show these associations, results have to be considered carefully as the complete case analyses may entail biased results due to potential selective drop-out (Bell et al. 2013). Further, there are also studies that did find different results indicating that low support from a superior was predictive of long-term sickness absence due to a psychiatric diagnosis (more than eight weeks; (Foss et al. 2010) or that find only non-significant associations between support and sickness absence, as the meta-analysis by Duchaine et al. (2020), for example. Thus, besides characteristics of study populations and sick leave duration, also the heterogeneity of assessed constructs can contribute to these mixed findings.

Only in sensitivity analyses considering complete cases, it was observed that possibilities for development predicted higher sickness absence days within the follow-up period (i.e., six months after intervention). However, we did not observe multicollinearity according to the variance inflation factor (VIF). In separate analyses controlling for confounding variables and a single psychosocial working condition scale, no significant association was found between possibilities for development and sickness absence days (ESM_1, ESM_2). This suggests, that this finding may be due to chance occurrence and would warrant further investigation to confirm its validity.

The lack of an association between demands and sickness absence days has also been seen in other studies investigating sickness absence due to depressive disorders (Clumeck et al. 2009; Inoue et al. 2010) or in a study investigating psychosocial factors and sickness absence in general (Niedhammer et al. 1998). For the present study, it seems that demands may be less relevant in terms of risks for subsequent sickness absence days among employees with symptoms of CMDs. However, there are also results from a meta-analysis indicating that high demands are prospectively associated with sickness absence due to a diagnosed mental disorder (Duchaine et al. 2020) or other results showing positive but also no associations for some studies (de Vries et al. 2018). Thus, these associations seem to change and look differently, depending on the study population, i.e., depending on whether study participants already had symptoms or whether a new development of CMD was investigated (as this was the case for associations between demands and sickness absence in (Duchaine et al. 2020). In light of the job-demand-control-support model, having control at work seemed to be more important for sickness absence in our study population, than the perception of demands at work.

We tested a number of additional scales assessing social relations and leadership quality, but did not observe any further associations with sickness absence days. It is important to mention that although we checked for multicollinearity with VIF, there may be high correlations between the variables assessing social relations. However, also separate analyses for every single scale did not show significant associations with sickness absence days in our study sample (ESM_1, ESM_2). While we have not found any association between recognition and sickness absence days, for example, other findings using the similar reward component of the effort-reward imbalance model, have shown increased risks for sickness absence due to mental health problems among men only, for low rewards (Ndjaboué et al. 2014). In accordance with our null findings for an association between sense of community and sickness absence days, another study among a representative sample of the French working population did also not find associations between sense of community and the number of sickness days (Bertrais et al. 2023). That study also showed that psychosocial working conditions were more likely to predict the cases of sickness absence than the number of sickness absence days (Bertrais et al. 2023). However, only in complete case analyses (not in single analyses), we observed a negative association between sense of community and sickness absence days for the follow-up period (ESM_3), which would thus go along with findings in the literature (Bertrais et al. 2023).

### Strengths and limitations

This is one of the first studies that investigated whether psychosocial working conditions predicted sickness absence days among employees reporting symptoms of CMDs in Germany. Thus, results for this specific study sample cannot be generalized onto other countries due to different labor laws, labor market situations or social insurance systems. Future research should aim to determine factors leading to potentially different associations (e.g., payment change from continued payment to sick pay, which countries have a staged return-to-work process).

It can be regarded as a strength that we have conducted prospective analyses covering more than one period and that we have chosen to investigate a range of different psychosocial working conditions. In contrast to many other studies, we have investigated any sickness absence days, and did not restrict our analyses to only long-term sickness absence. It has been shown that psychosocial work factors were associated with both, shorter and longer periods of sickness absence in Danish employees, for example (Thorsen et al. 2021).

There are several limitations that have to be considered when interpreting our results. As the analyses are secondary to the RCT (Weber et al. 2021), we did not perform additional a-priori power analyses but present rate ratios and confidence intervals. All measurements are based on self-report, which could introduce common method variance (Podsakoff et al. 2003). However, in regard of sickness absence, it has been shown that self-reported sickness absence data can be similar to employer’s register-based sickness absence data (Voss et al. 2008). Quality controls were included, in which unreasonable sickness absence days (i.e., reporting of higher days than the possible time period) were double checked with the participants and baseline sickness absence days were double checked by the study psychotherapists.

We cannot exclude that the perception of psychosocial working conditions, assessed at baseline, has changed over time for the participants. Especially, as half of the employees took part in an intervention which was aimed at reducing symptoms of CMDs by understanding and dealing with the impact of psychosocial working conditions on mental health, it may be possible that the perception of working conditions has changed. If perceptions of working conditions have changed over the investigated period, the results at hand may be misleading. Future analyses would need to clarify this. In addition, this may indicate that the participants were more interested in improving their mental health status (as they participated in an RCT in which they received psychotherapeutic consultations), than a general working population with CMD symptoms. Results of analyses covering only the follow-up period (i.e., T2) have to be considered carefully as we have observed convergence issues when including the independent variables into the analyses. Thus, the addition of further variables did not result in increased variance but rather showed that the model became unstable, probably due to the complexity and broad range of psychosocial working conditions. Further, all our models show generally low model fit values, indicating that there may be other more important predictors for sickness absence days among our study population (Montano 2020).

As we have observed some additional associations in sensitivity analyses considering complete cases (ESM_3), it is important to mention that the complete case analyses may be biased due to selective dropout and should be considered carefully. For example, we observed that, on average, sickness absence days at baseline were lower among the participants who reported sickness absence days for all three time periods compared to participants with missing data for any sickness absence days (difference of about four days, data not shown), indicating that there may be a selective drop-out of participants with higher sickness absence days at baseline. Something similar was checked and observed for some of the psychosocial working conditions: More favorable working conditions (for recognition, dissolution, unfair treatment and degrees of freedom) were found among the participants who reported sickness absence days at all three time points, indicating that participants with less favorable working conditions have dropped out of the study.

### Implications

As we have investigated which psychosocial working conditions are predictive of subsequent sickness absence days at different time points, it would also be of interest to investigate in the future, if changes in the perception of working conditions (possibly due to the intervention) and in objective working conditions may contribute to different risks of sickness absence days. However, as we could not observe that the investigated broad range of psychosocial working conditions had a great effect on sickness absence days, it seems rather unlikely that calculated changes would show very different results. In line with this thought, there are studies available, which showed for example, that adverse changes in psychosocial working conditions increased the risk for sickness absence only slightly, and that favorable changes in working conditions were not associated with sickness absence at all (Saastamoinen et al. 2014). Other findings suggested that changes in sickness absence are not affected by changes in perception of psychosocial working conditions in the Finnish Food Industry (Siukola et al. 2011). Thus, there may be other factors (such as previous sickness absence or age in our case), which are more important in regard of sickness absence days (Montano 2020). In light of these, Montano (2020) also considers the relevance of the effects of psychosocial working conditions, i.e., how individuals react to and cope with these, as well as the subjective health status and a range of moderators or mediators for the likelihood of sickness absence. Among our study population, factors outside of the working environment, such as private conflicts or family conflicts may be more relevant for sickness absence than psychosocial working conditions. For example, in our sample, 71% of the participants reported to participate in the PT-A due to work-related stress and personal problems (Rothermund-Nassir et al. 2025). This may explain that for sickness absence other factors were more relevant next to the psychosocial working conditions. Nevertheless, improving influence for employees (regarding amount of work and decisions affecting work) may be a good opportunity to reduce sickness absence days, as by this a greater flexibility may help to deal with the work tasks according to one’s own workability, and has to be tested in future research (Helgesson et al. 2023). Repeating the analyses within a representative sample may also be helpful for interpretation of our results. As we performed the study with employees who took part in a RCT mostly via the company, it may be that the participants had better working conditions that allowed them to participate than a general working population with CMD symptoms. It may also be possible that they have a more active health behavior as they were actively seeking for support to cope with their symptoms. Another promising future research idea would be to investigate the effects for different occupational sectors. As working conditions may differ in certain occupations (for example in healthcare, in crafts or in administration the most stressful conditions may differ), there may be different magnitudes of effects for working conditions on sickness absence days among employees with CMD. Results may be relevant to find suitable starting points for interventions to reduce sickness absence days for these occupational groups, as well.

## Conclusion

This study investigated whether a number of psychosocial working conditions are predictors of sickness absence days in a sample of employees reporting symptoms of CMDs and participating in a randomized controlled trial investigating the effectiveness of psychotherapeutic consultation at work (PT-A). Results showed that employees who reported higher influence at work had lower risks of sickness absence days at nine and 15 months. Higher dissolution was associated with a lower risk of sickness absence days during the follow-up period. However, most of the psychosocial working conditions we considered were not predictive of sickness absence days, suggesting that other factors may be more relevant for sickness absence days among employees with symptoms of CMD in Germany.

## Supplementary Information

Below is the link to the electronic supplementary material.

Supplementary Material 1

Supplementary Material 2

Supplementary Material 3

## Data Availability

The datasets generated and/or analyzed during the current study are not publicly available due to data protection regulations, but are available from the study center director on reasonable request.
