# Supplementary material for: The relationship between psychosocial working conditions and sickness absence days among employees reporting symptoms of common mental disorders in Germany
Source: Int Arch Occup Environ Health. 2026 Mar 11;99(3):17. doi: 10.1007/s00420-026-02205-7 (PMC12979410; doi:10.1007/s00420-026-02205-7)
Supplement: Supplementary file 1 — Supplementary Material 1 [file 420_2026_2205_MOESM1_ESM.pdf]

Journal: International Archives of Occupational and Environmental Health

The relationship between psychosocial working conditions and sickness absence days among employees reporting symptoms of common mental disorders in Germany

Meike Heming[1*], Florian Angerer, Christoph Kröger, Gianni Lidolt, Nicole R. Hander, Eva Rothermund, Harald Gündel, Nadine Mulfinger, Ute Schröder, Uta Wegewitz, Regina Herold & Peter Angerer

[1] Institute of Occupational, Social, and Environmental Medicine, Centre for Health and Society, Medical Faculty and University Hospital Düsseldorf, Heinrich-Heine-University Düsseldorf, Moorenstraße 5, 40225 Düsseldorf, Germany

Correspondence: Meike.Heming@hhu.de

Table S1. Results for separate negative binomial regression analyses estimating the association between each psychosocial working condition and sickness absence days for imputed data (n = 529).

	**Sickness absence days**											
	**T1 (n = 529)**				**T1 + T2 (n = 529)**				**T2 (n = 529)**			
		95% CI[a]				95% CI[a]				95% CI[a]		
	RR[b]	lower	upper	p-value	RR[b]	lower	upper	p-value	RR[b]	lower	upper	p-value
**Demands**												
Quantitative demands	1.00	0.99	1.01	0.753	1.00	0.99	1.01	0.793	1.00	0.99	1.01	0.314
Emotional demands	1.00	0.99	1.00	0.382	1.00	0.99	1.00	0.325	1.00	0.99	1.00	0.892
Dissolution	1.00	0.99	1.01	0.811	1.00	0.99	1.00	0.400	**0.99**	**0.99**	**1.00**	**0.050**
**Influence and possibilities for development**												
Influence at work	**0.99**	**0.99**	**1.00**	**0.014**	**0.99**	**0.99**	**1.00**	**0.020**	1.00	0.99	1.00	0.603
Degrees of freedom	1.00	1.00	1.01	0.548	1.00	1.00	1.01	0.596	1.00	1.00	1.01	0.412
Possibilities for development	1.00	0.99	1.01	0.807	1.00	0.99	1.01	0.960	1.01	0.99	1.01	0.256
**Social relations and leadership**												
Quality of leadership	1.00	0.99	1.00	0.249	1.00	0.99	1.00	0.336	1.00	0.99	1.01	0.849
Support at work	1.00	0.99	1.01	0.864	1.00	0.99	1.01	0.853	1.00	0.99	1.01	0.634
Sense of community	1.00	0.99	1.01	0.669	1.00	0.99	1.00	0.334	1.00	0.99	1.01	0.556
Unfair treatment	1.00	1.00	1.01	0.112	1.00	1.00	1.01	0.108	1.00	1.00	1.01	0.952
Trust and justice	1.00	0.99	1.01	0.422	1.00	0.99	1.01	0.787	1.00	0.99	1.02	0.573
Recognition	1.00	0.99	1.00	0.258	1.00	0.99	1.00	0.479	1.00	0.99	1.01	0.779

[a] 95 % confidence interval.

[b] Rate ratio.

Each line represents a single analysis. Analyses are adjusted for sickness absence days (either T0 or T1), sex, age, treatment group, occupational position, depression severity (either T0 or T1), anxiety symptoms (either T0 or T1), somatic symptoms (either T0 or T1).
