# Supplementary material for: The relationship between psychosocial working conditions and sickness absence days among employees reporting symptoms of common mental disorders in Germany
Source: Int Arch Occup Environ Health. 2026 Mar 11;99(3):17. doi: 10.1007/s00420-026-02205-7 (PMC12979410; doi:10.1007/s00420-026-02205-7)
Supplement: Supplementary file 2 — Supplementary Material 2 [file 420_2026_2205_MOESM2_ESM.pdf]

Journal: International Archives of Occupational and Environmental Health

The relationship between psychosocial working conditions and sickness absence days among employees reporting symptoms of common mental disorders in Germany

Meike Heming[1*], Florian Angerer, Christoph Kröger, Gianni Lidolt, Nicole R. Hander, Eva Rothermund, Harald Gündel, Nadine Mulfinger, Ute Schröder, Uta Wegewitz, Regina Herold & Peter Angerer

[1] Institute of Occupational, Social, and Environmental Medicine, Centre for Health and Society, Medical Faculty and University Hospital Düsseldorf, Heinrich-Heine-University Düsseldorf, Moorenstraße 5, 40225 Düsseldorf, Germany

Correspondence: Meike.Heming@hhu.de

Table S2. Results for separate negative binomial regression analyses estimating the association between each psychosocial working condition and sickness absence days for complete cases.

	**Sickness absence days**											
	**T1 (n = 377)**				**T1 + T2 (n = 320)**				**T2 (n = 313)**			
		95 % CI[a]				95 % CI[a]				95 % CI[a]		
	RR[b]	lower	upper	p-value	RR[b]	lower	upper	p-value	RR[b]	lower	upper	p-value
**Demands**												
Quantitative demands	1.00	1.00	1.01	0.438	1.00	0.99	1.01	0.432	1.00	0.99	1.00	0.243
Emotional demands	1.00	0.99	1.00	0.288	1.00	0.99	1.00	0.663	1.00	1.00	1.01	0.403
Dissolution	1.00	0.99	1.01	0.915	1.00	0.99	1.00	0.235	1.00	1.00	1.01	0.403
**Influence and possibilities for development**												
Influence at work	**0.99**	**0.98**	**1.00**	**0.012**	1.00	0.99	1.00	0.215	1.00	0.99	1.01	0.404
Degrees of freedom	1.00	1.00	1.01	0.430	1.00	0.99	1.00	0.645	1.00	0.99	1.00	0.141
Possibilities for development	1.00	0.99	1.01	0.789	1.00	0.99	1.01	0.809	1.00	1.00	1.01	0.325
**Social relations and leadership**												
Quality of leadership	1.00	0.99	1.00	0.273	1.00	0.99	1.00	0.574	1.00	0.99	1.01	0.958
Support at work	1.00	0.99	1.01	1.000	1.00	1.00	1.01	0.384	1.00	0.99	1.01	0.655
Sense of community	1.00	0.99	1.01	0.931	1.00	0.99	1.00	0.432	0.99	0.98	1.00	0.127
Unfair treatment	1.01	1.00	1.01	0.061	1.00	1.00	1.01	0.351	1.00	1.00	1.01	0.524
Trust and justice	1.00	0.99	1.00	0.232	1.00	0.99	1.01	0.702				
Recognition	1.00	0.99	1.00	0.071	1.00	0.99	1.00	0.302	1.00	0.99	1.01	0.768

[a] 95 % confidence interval.

[b] Rate ratio.

Each line represents a single analysis. Analyses are adjusted for sickness absence days (either T0 or T1), sex, age, treatment group, occupational position, depression severity (either T0 or T1), anxiety symptoms (either T0 or T1), somatic symptoms (either T0 or T1).
