# Supplementary material for: The relationship between psychosocial working conditions and sickness absence days among employees reporting symptoms of common mental disorders in Germany
Source: Int Arch Occup Environ Health. 2026 Mar 11;99(3):17. doi: 10.1007/s00420-026-02205-7 (PMC12979410; doi:10.1007/s00420-026-02205-7)
Supplement: Supplementary file 3 — Supplementary Material 3 [file 420_2026_2205_MOESM3_ESM.pdf]

Journal: International Archives of Occupational and Environmental Health

The relationship between psychosocial working conditions and sickness absence days among employees reporting symptoms of common mental disorders in Germany

Meike Heming[1*], Florian Angerer, Christoph Kröger, Gianni Lidolt, Nicole R. Hander, Eva Rothermund, Harald Gündel, Nadine Mulfinger, Ute Schröder, Uta Wegewitz, Regina Herold & Peter Angerer

[1] Institute of Occupational, Social, and Environmental Medicine, Centre for Health and Society, Medical Faculty and University Hospital Düsseldorf, Heinrich-Heine-University Düsseldorf, Moorenstraße 5, 40225 Düsseldorf, Germany

Correspondence: Meike.Heming@hhu.de

Table S3. Results for negative binomial regression analyses estimating the association between psychosocial working conditions and sickness absence days for complete cases.

	Sickness absence days											
	T1 (n = 377)				T1+T2 (n = 320)				T2 (n = 313)			
		95 % CI[a]				95 % CI[a]				95 % CI[a]		
	RR[b]	lower	upper	p-value	RR[b]	lower	upper	p-value	RR[b]	lower	upper	p-value
Sickness absence days T0 or T1	**1.01**	**1.01**	**1.02**	**0.000**	**1.01**	**1.01**	**1.02**	**0.000**	**1.01**	**1.008**	**1.015**	**0.000**
Sex	0.82	0.57	1.18	0.246	0.91	0.64	1.29	0.560	1.12	0.744	1.692	0.580
Age	**1.03**	**1.01**	**1.05**	**0.000**	**1.04**	**1.02**	**1.05**	**0.000**	1.02	0.995	1.034	0.114
Treatment group	1.11	0.80	1.54	0.517	1.01	0.74	1.38	0.932	0.92	0.624	1.344	0.652
Occupational position	0.84	0.53	1.30	0.401	0.81	0.52	1.24	0.310	0.76	0.433	1.295	0.285
Depression severity T0 or T1	**1.08**	**1.03**	**1.13**	**0.001**	**1.05**	**1.00**	**1.09**	**0.045**	1.05	0.984	1.121	0.145
Anxiety symptoms T0 or T1	1.01	0.89	1.14	0.906	1.05	0.93	1.19	0.359	0.96	0.806	1.155	0.656
Somatic symptoms T0 or T1	0.99	0.96	1.03	0.688	1.02	0.98	1.06	0.322	1.04	0.99	1.1	0.093
**Demands**												
Quantitative demands	1.00	0.99	1.01	0.599	1.00	0.99	1.01	0.588	0.99	0.982	1.004	0.148
Emotional demands	1.00	0.99	1.00	0.485	1.00	0.99	1.00	0.594	1.00	0.995	1.008	0.586
Dissolution	1.00	1.00	1.01	0.539	1.00	0.99	1.00	0.310	**0.99**	**0.984**	**0.999**	**0.027**
**Influence and possibilities for development**												
Influence at work	**0.99**	**0.98**	**1.00**	**0.024**	1.00	0.99	1.01	0.430	1.00	0.985	1.005	0.336
Degrees of freedom	1.00	1.00	1.01	0.547	1.00	0.99	1.00	0.228	1.00	0.988	1.003	0.241
Possibilities for development	1.00	0.99	1.01	0.567	1.00	0.99	1.01	0.510	**1.01**	**1**	**1.022**	**0.046**

**Social relations and leadership**												
Quality of leadership	1.00	0.99	1.01	0.545	0.99	0.98	1.00	0.179	0.99	0.982	1.005	0.235
Support at work	**1.01**	**1.00**	**1.02**	**0.038**	**1.01**	**1.00**	**1.03**	**0.004**	1.01	0.999	1.024	0.074
Sense of community	1.00	0.99	1.01	0.867	0.99	0.98	1.00	0.071	**0.99**	**0.979**	**0.999**	**0.035**
Unfair treatment	1.01	1.00	1.01	0.176	1.00	1.00	1.01	0.322				
Trust and justice	1.00	0.99	1.01	0.973	1.00	0.99	1.01	0.588				
Recognition	1.00	0.99	1.01	0.429	1.00	0.99	1.01	0.383	1.00	0.991	1.008	0.942
**Model Fit**	AIC [c]	BIC [d]		MC Fadden's R^2	AIC [c]	BIC [d]		MC Fadden's R^2	AIC [c]	BIC [d]		MC Fadden's R^2
	3196.862	3283.371		0.027	3086.621	3169.524		0.026	2218.217	2293.141		0.043

[a] 95 % confidence interval.

[b] Rate ratio.

[c] Akaike information criterion.

[d] Bayesian information criterion.
